# Lithium Slag and Solid Waste-Based Binders for Cemented Lithium Mica Fine Tailings Backfill

**DOI:** 10.3390/ma16247572

**Published:** 2023-12-09

**Authors:** Jiafeng Li, Jinsong Huang, Yali Hu, Daopei Zhu

**Affiliations:** 1 School of Civil and Surveying & Mapping Engineering, Jiangxi University of Science and Technology, Ganzhou 341000, China; 6720211196@mail.jxust.edu.cn (J.L.); 6720211194@mail.jxust.edu.cn (Y.H.); 2 Zhejiang Zhipu Engineering Technology Co., Ltd., Huzhou 313000, China

**Keywords:** lithium slag, industrial solid wastes, lithium mica fine tailings, cementitious material, uniaxial compression test

## Abstract

To mitigate the adverse effects of fine-grained lithium mica tailings and other solid wastes generated from the extraction of lithium ore mining, as well as the limitations of traditional cement-based binders for lithium mica fine tailings, this study explores the feasibility of using a binder composed of ordinary Portland cement, lithium slag, fly ash, and desulfurization gypsum to stabilize lithium fine tailings into cemented lithium tailings backfill. Compared with traditional cementitious binders, an extensive array of experiments and analyses were conducted on binders formed by various material proportion combinations, employing uniaxial compressive strength tests, microstructural morphology, grayscale analyses, and flowability tests. The results show the following: (1) In this study, an LSB binder exhibiting superior mechanical properties compared to traditional cementitious binders was identified, with an optimal OPC:LS:FA:DG ratio of 2:1:1:1. (2) In the context of cemented lithium mica fine tailings, the LSB-CLTB material exhibits higher unconfined compressive strength and lower self-weight compared to OPC-CLTB materials. At a binder content of 10 wt%, the UCS values achieved by the LSB-CLTB material at curing periods of 7 days, 14 days, and 28 days are 0.97 MPa, 1.52 MPa, and 2.1 MPa, respectively, representing increases of 40.6%, 34.5%, and 44.8% over the compressive strength of OPC-based materials under the same conditions. (3) The LSB binder not only exhibits enhanced pozzolanic reactivity but also facilitates the infilling of detrimental pores through its inherent particle size and the formation of AFt and C-(A)-S-H gels via hydration reactions, thereby effectively improving the compressive strength performance of fine-grained tailings backfill.

## 1. Introduction

The rapid development of the technology sector and rising global energy demand have made the widespread utilization of lithium resources a key priority [1,2,3]. However, as the lithium mining industry continues to expand, associated issues are becoming increasingly prominent, particularly the handling of ore tailings generated during the extraction and processing of lithium mica ores [4,5]. As mineral resources are increasingly exploited, high-grade resources are decreasing. To obtain more mineral resources, the particle size of the raw ore must be finer, and high-efficiency grinding technology is required for the production of large quantities of ultrafine tailings [6]. Currently, underground mines, especially small- and medium-sized mines, employ a classification process for the tailings produced from mineral resource extraction. Coarse tailings resulting from this classification process are used for underground backfilling, while the remaining fine tailings are stored within tailings [7]. The small particle size and large specific surface area of fine-grained tailings can easily affect the stability of tailing dams and cause “dangerous warehouses”, which also incur storage costs for mines [8]. Currently, the most common treatment method for tailings is cement-based filling [9,10]. However, the cost of cement accounts for approximately 70% of the total filling cost in cement-based filling mining and has become a key limiting factor [11,12]. Relevant studies have shown that cement has the disadvantages of poor cementation ability, low filling strength, and high hydration heat for fine-grained tailings [13,14]. Additionally, the production and use of cement in various industries result in significant carbon dioxide emissions, making it one of the largest global sources and impacting the environment [15,16]. Therefore, finding a suitable cementitious material for filling lithium mica fine-grained tailing aggregates is crucial for achieving safe, efficient, and sustainable, cost-effective mining operations. This is also a challenge faced by an increasing number of lithium resource mines.

Lithium slag (LS) is a byproduct of lithium production and processing. Each ton of lithium hydroxide produces 20–40 tons of LS, whereas each ton of lithium carbonate produces 10 tons of LS [17]. This underscores the significance of appropriately managing LS for sustainable development. Nonetheless, it is estimated that approximately 90% of LS treatment methods consist of direct disposal onto the surface, leading to considerable resource waste and environmental pollution [18]. Previous studies have shown that LS has high pozzolanic activity owing to its rich oxide or mineral phases, such as silica, which makes it beneficial for partially replacing cement and enhancing compressive strength [19]. Additionally, fly ash (FA) and desulfurization gypsum (DG), as solid wastes inevitably generated during industrial production and mineral resource processing, have also been extensively studied and developed by scholars, both domestically and internationally. Filho et al. used FA and hydrated lime in cement and found that they could improve the microstructure of the slurry, effectively enhancing the durability of concrete [20]. Janardhan et al. improved the mechanical properties of geopolymers by adding an appropriate proportion of ground-granulated blast-furnace slag and FA to cement [21]. Wan et al. developed a flue gas desulfurization gypsum-based binder for soil stabilization and suggested that the flue gas desulfurization gypsum-based binder exhibits promising pozzolanic activity in the later stages [22]. Jiang et al., through experiments and analyses, concluded that the addition of desulfurization gypsum effectively facilitates the formation of ettringite, which is beneficial for enhancing the early strength, high reliability, and durability of concrete [23].

In summary, the binder formed by mixing LS, FA, and DG in ordinary Portland cement (OPC) is theoretically a new type of binder that can substantially improve the activity of volcanic ash, has strong cementation ability, and is conducive to improving the compressive strength of backfilling [24,25,26]. The purpose of this study is to explore the feasibility of LS, FA, and DG for cementing lepidolite fine-grained tailings for backfilling. The high domestic and international demand for lithium resources has resulted in large amounts of LFTs during the extraction and processing of lithium ores. To solve this issue, we propose to combine cement and other solid wastes to prepare cemented lithium mine filling materials. This solution not only reduces the problems of environmental pollution and surface damage caused by mining but also significantly decreases the cost of mine filling operations. This study offers a new approach for the comprehensive utilization of LFTs and provides a valuable reference for addressing the issues related to lithium resource supply and solid waste management.

## 2. Materials and Methods

### 2.1. LS Composite Solid Waste Cementitious Material

**Figure 1:**
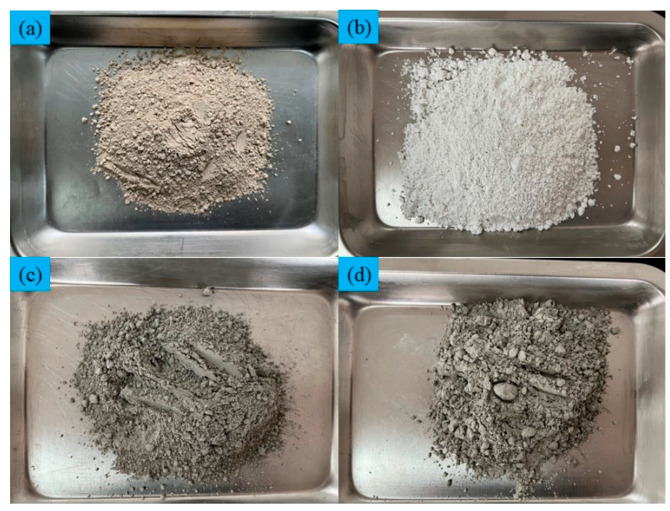
Materials: (**a**) lithium slag, (**b**) desulfurization gypsum, (**c**) ordinary Portland cement, and (**d**) fly ash.

**Figure 2:**
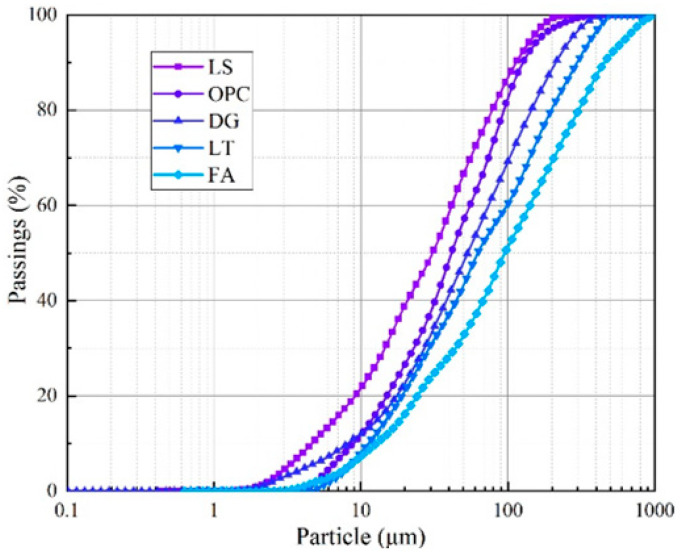
Cumulative particle size distribution curve.

The LS composite solid waste cementitious material, as shown in Figure 1, is mainly composed of LS, FA, DG, and OPC. The OPC selected was PO42.5 (equivalent to CEM I-42.5), produced by Henan Xinxiang Yanjin Jianye Cement Manufacturing Company, Xinxiang, China. The LS was sourced from a lithium mine in Yichun, China. Following rudimentary pretreatment, the samples were pulverized and desiccated. The samples prepared as pellets using a high-pressure pellet press were analyzed using an X-ray fluorescence spectrometer (ZSX Primus III+, Shenzhen Hua General Technology Co., Ltd., Shenzhen, China) to record characteristic X-rays and spectral data. Additionally, the composition and phase content were analyzed using an XRD diffractometer (Bruker D8 ADVANCE, Shanghai Jiezhu Instrument & Equipment Co., Ltd., Shanghai, China). Before these analyses, each material was thoroughly dispersed in a wet medium. Subsequently, a laser particle size analyzer (Malvern Mastersizer 3000, Shanghai Sibaiji Instrument System Co., Ltd., Shanghai, China) was employed to conduct a particle size analysis by emitting a laser through the particle suspension in the sample, as illustrated in Figure 2. The experimental procedures were consistent with the methods described in a reference study [27].

**Figure 3:**
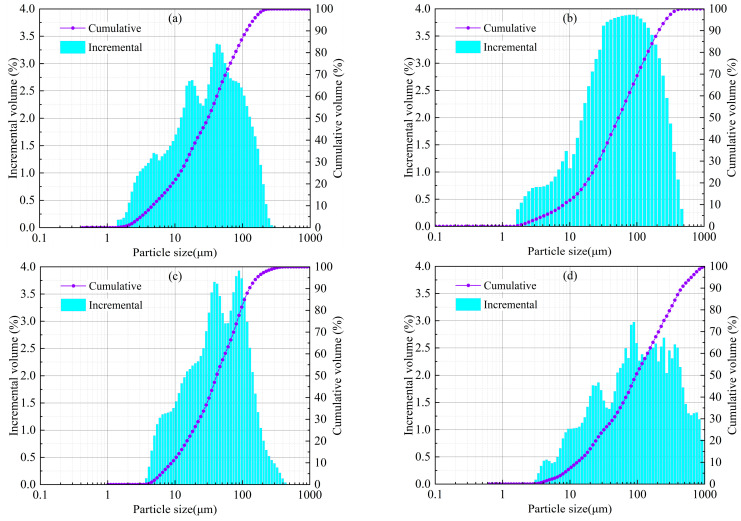
Particle size distribution of lithium slag composite solid waste cementitious material: (**a**) LS, (**b**) DG, (**c**) OPC, and (**d**) FA.

In terms of physical properties, Table 1 and Figure 3 provide information on the particle size parameters of each material. It can be seen from the chart that LS has the finest particle size, with 41.1% of fine particles less than 20 μm, and the average particle size d (bar) is 34.58 μm. The particle size distributions of the OPC and DG are relatively close. The contents of fine particles less than 20 μm are 26.6% and 24.6%, respectively, and the average particle size d (bar) values are 42.18 μm and 53.04 μm, respectively. FA has the coarsest particle size, with 18% of fine particles less than 20 μm, and the average particle size d (bar) is 97.63 μm. The above results show that LS is the main source of fine particles in the LS composite solid waste cementitious material. Furthermore, as listed in Table 1, based on the principles of soil mechanics, to accurately analyze the particle size distribution of each material, the non-uniformity coefficient C_u_ and curvature coefficient C_c_ can be used to evaluate the particle size distribution. When the non-uniformity coefficient C_u_ ≥ 5 and 1 < C_c_ < 3, the particles have good gradation and are relatively easy to compact [28]. The raw materials of the LSB meet this requirement, which indicates that the selected materials of the LSB are easy to compact. This property is beneficial for improving the compressive strength of the CLTB.

**Figure 4:**
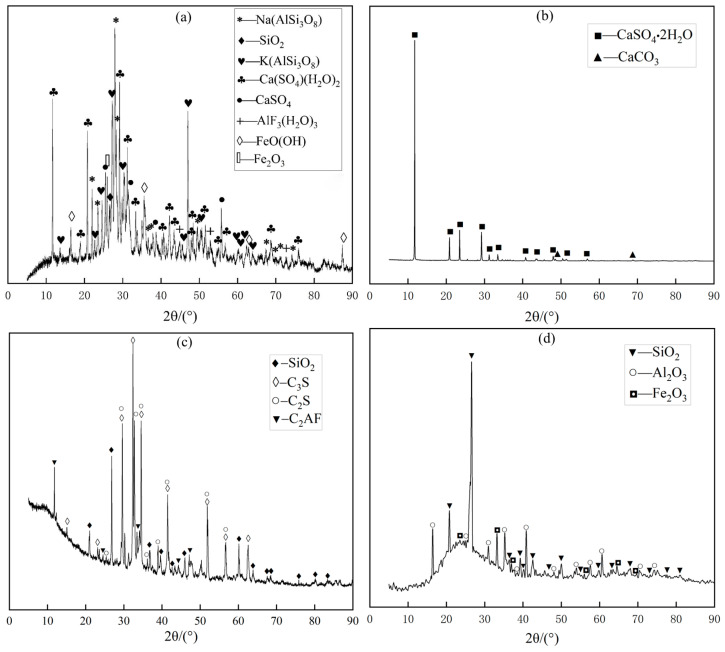
XRD patterns of lithium slag composite solid waste cementitious material: (**a**) LS, (**b**) DG, (**c**) OPC, and (**d**) FA.

The chemical compositions obtained from the XRF and XRD test results are displayed in Table 2 and Figure 4. From the chart, the main components of LS are CaO, SiO_2_, and Al_2_O_3_, while FA mainly comprises SiO_2_, Al_2_O_3_, and Fe_2_O_3_. The main component of DG is CaSO_4_⋅2H_2_O, while OPC mainly consists of CaO, SiO_2_, and Al_2_O_3_. This indicates that LS, FA, and DG have potential cementitious activity and can be possible substitutes for cementitious materials [29]. A pH detector (with an accuracy of ±0.01) was used to measure the acidity and alkalinity of the materials. The LS used in this study was a waste residue produced during lithium carbonate production with a pH of 5.5. Alkaline conditions can effectively stimulate the pozzolanic activity of LS. Weakly alkaline DG (pH = 7.8) and highly alkaline OPC and FA (pH = 12.8 and 11.6, respectively) can provide a favorable alkaline environment for improving the hydration performance of LS, so that the CLTB has a higher compressive strength [30].

### 2.2. Cemented Aggregate Material

**Figure 5:**
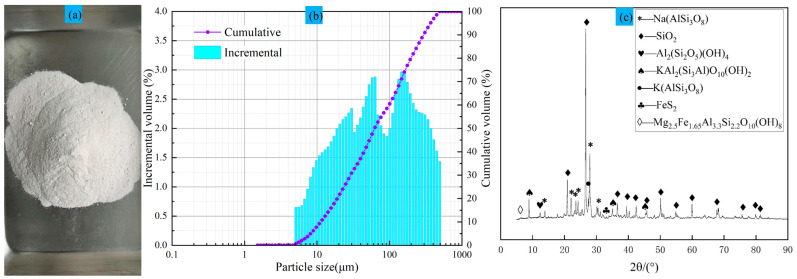
(**a**) LFT cemented filling aggregate. (**b**) Particle size distribution of LFT. (**c**) XRD pattern of LFT.

**Figure 6:**
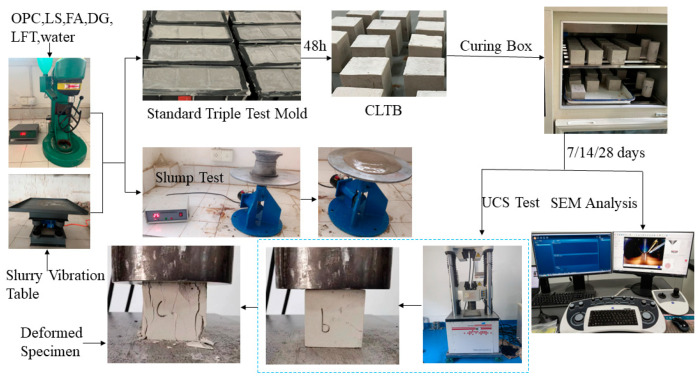
Diagram of experimental procedures. (The c, b marks in the test block only represent the test block label.)

The cemented aggregate material was LFT from a lithium mine in Yichun, China, as illustrated in Figure 5a. The LFT particle size test results are shown in Figure 5b, while the XRD test results are presented in Figure 6. It can be observed from the charts that the content of fine particles less than 20 μm in the LFT is 21.9%, and the average particle size d (bar) is 63.27 μm. The main components of cement are albite, quartz, and kaolin. Based on the mineral composition and previous studies, the quartz and kaolin present in the LFT do not have adverse effects on the strength of the CLTB [31,32]. Similarly, the near-neutral inert material LFT (pH = 6.7) does not adversely affect the LS composite solid waste cementitious material. It is worth noting that lepidolite fine tailings have a C_c_ of less than 1, indicating poor gradation of the LFT particles.

### 2.3. Experimental Scheme

(1) Cementation performance of LS composite solid waste cementitious material

The cementation properties of LS composite solid waste cementitious materials were investigated by preparing samples with different proportions of OPC, LS, FA, and DG. Based on the results after trial tests, samples with different proportions of OPC, LS, FA, and DG were selected and prepared for the uniaxial compression tests (Table 3). Comparisons were made with specimens solely comprising cement-based binders and specimens exclusively using latent hydraulic materials such as LS and FA. The corresponding OPC:LS:FA:DG ratios for these were 5:0:0:0, 0:5:0:0, and 0:0:5:0, respectively. In the specimens where only DG was added, no significant compressive strength was exhibited in the early stages; hence, these specimens were not subjected to experimental comparison. The specimen with the highest compressive strength corresponded to the optimal proportion of OPC:LS:FA:DG, which is considered the best mix ratio for lithium slag-based composite solid waste binder (LSB).

(2) Experimental study on the cementation of LFT with LSB

LFTs are widely recognized as unsuitable backfill aggregates owing to issues such as fine particle size, low compressive strength of cemented backfill, poor slurry fluidity, and high cementitious material content. To further investigate the capability of LSB binders in cementing fine-grained lithium mica tailings for backfilling purposes, compressive strength tests were conducted on CLTB specimens with varying LSB mass fractions (5%, 10%, 15%, and 20%), controlling the content of the binder. Concurrently, CLTB specimens utilizing a traditional OPC binder at equivalent mass fractions were compared to analyze the resultant variations in compressive strength. This study also explores whether LSB-CLTB meets the practical engineering requirements.

(3) Study on the fluidity test of LSB-CLTB

The two most important performance indicators of backfilling used in actual projects are the backfilling strength and fluidity of the backfilling slurry. The fluidity of a slurry refers to its ability to flow under the action of its own weight or pumping pressure, which can produce either positive or negative effects. It is an important property for determining whether the slurry can be safely and efficiently transported to goafs for filling. Moreover, LS may exhibit significant water absorption owing to its relatively fine particle size, which has a high surface activity and adsorption capacity. This can affect the fluidity of the CLTB slurry to a certain extent [33]. Considering the accuracy and practical operability of the filling slurry fluidity test, this study used the “cement mortar fluidity measurement method” (GB/T 2419-2005) to measure the fluidity of the filling slurry using a jump table, a truncated cone die, and a vernier caliper [34]. The actual backfilling transportation pipeline in a lithium mine in Yichun, China, requires a slump of 190–270 mm or a fluidity of more than 200 mm. Therefore, only the results that met this fluidity requirement were subsequently studied and analyzed [35,36].

### 2.4. Experimental Process

First, the physical and chemical properties of OPC, LS, FA, DG, and LFTs were analyzed using a laser particle size analyzer, an X-ray diffractometer, and other instruments. Subsequently, the optimal ratio of OPC:LS:FA:DG to form an LSB was determined through numerous experimental comparisons. Then, backfill test blocks formed by the LSB-CLTB were subjected to uniaxial compression tests, fluidity tests, SEM imaging, and gray value analyses. The backfill mechanical properties, microstructural characteristics, and the hydration reaction gelation mechanism of LSB-CLTB were discussed and analyzed. As illustrated in Figure 6, the test process involves sample preparation, fluidity testing, treatment in a constant temperature and humidity curing box, an uniaxial compression test, an XRD analysis, and SEM imaging.

(1) Specimen preparation

According to the experimental scheme and calculation ratio, the amount of each material was weighed to a precision of 0.01 g. To minimize the impact of the sample demolding process on the experimental results, a small amount of release agent was applied to the inner surface of the mold 24 h prior to the experiment. First, the materials without water were pre-stirred for 2 min to make the distribution of each material more uniform, and then the specified quality of water was added and stirred for 3 min. A portion of the uniformly stirred filling slurry was taken for the slurry fluidity test, while the rest was poured into a triple test mold (70.7 × 70.7 × 70.7 mm) coated in advance with a release agent. The test mold was placed in a symmetrical position relative to the centerline of the slurry-shaking table, and bubbles were eliminated according to the specified vibration parameters to ensure the compactness of the filling slurry in the test mold. Finally, initial curing was carried out at room temperature for 48 h, and then the cured samples were placed in a standard constant temperature and humidity curing box (SHBY-40B type) with a temperature of 20 ± 1 °C and a relative humidity of more than 90% until they reached the specified age.

(2) Slump test

The fluidity test was conducted using a truncated cone die with a height of 0.5 mm, an inner diameter of 70 mm ± 0.5 mm at the upper opening, and an inner diameter of 100 mm ± 0.5 mm at the lower opening. Jump tables and vernier calipers were used to measure the fluidity of the filling slurry. First, the uniformly stirred filling slurry was placed into a truncated cone die in accordance with the standards. The die was then lifted while the jump table was started. The jumping process was completed 25 times within a period of 25 ± 1 s at a frequency of once per second. A vernier caliper was used to measure the diameters in two perpendicular directions on the bottom surface of the filling slurry, from which the average value was calculated and rounded to an integer value. This value represents the fluidity of the filling slurry. The entire fluidity test process took approximately 6 min, from mixing water with the filling slurry until the diffusion diameter was measured. The fluidity tests for all samples were repeated three times to determine the slurry fluidity.

(3) Uniaxial compression test and XRD test

The uniaxial compression tests for the CLTB specimens were performed using an SHT4605 microcomputer-controlled electrohydraulic servo universal testing machine (Shenzhen Sansi Test Equipment Co., Ltd., Shenzhen, China). The results showed that the loading rate has an effect on the strength of the cemented backfill. When the loading rate was 0.5–1.0 mm/min, the deformation of the specimen was more uniform [37]. Therefore, a loading rate of 0.5 mm/min was used to test the samples for 7, 14, and 28 days. Similarly, all tests were repeated three times, and the average value of the three tests was considered the compressive strength of each sample. The results were accurate to within 0.01 MPa. The experimental procedures were consistent with the methods described in a reference study [38]. Simultaneously, the phase composition of the tested material samples was analyzed using an X-ray diffractometer(Bruker D8 ADVANCE, Shanghai Jiezhu Instrument & Equipment Co., Ltd., Shanghai, China), under an electrical load of 40 kV tube voltage and 40 mA current. The analysis was performed in the scanning range of 5° to 90° and at a scanning speed of 2°/min, utilizing the Kα line (1.54 Å) generated by a Cu target.

(4) SEM experiments

ZEISS Sigma 300 (Shenzhen Huatong General Technology Co., Ltd., Shenzhen, China) was employed to conduct the SEM tests. Internal test blocks from the CLTB samples after uniaxial compressive strength (UCS) testing were selected for SEM analysis, and samples with dimensions less than 1 cm in length, width, and height were prepared. To acquire stable and high-resolution SEM images, it is essential to ensure that the sample has good conductivity. Initially, the sample directly adheres to a conductive adhesive, followed by gold sputtering using a Quorum SC7620 sputter coater(Mycono Technology Co., Ltd., Shanghai, China). The gold sputtering process for each test sample is uniformly controlled at a duration of 120 s and a sputtering current of 10 mA. Subsequently, the particle morphology and pore structure of the CLTB samples were examined using the SEM. The experimental procedures were aligned with the methods described in our references [39,40]. Python (3.8.18) and Pycharm (2022.2.1) were then utilized to analyze the gray values of the SEM images. Functions NumPy, Matplotlib, OpenCV, and SciPy were employed to perform 3D image conversion based on gray values within a specific region. Finally, layered rendering was applied to facilitate the subsequent analysis.

## 3. Results and Discussion

### 3.1. Bonding Properties of Binders

**Figure 7:**
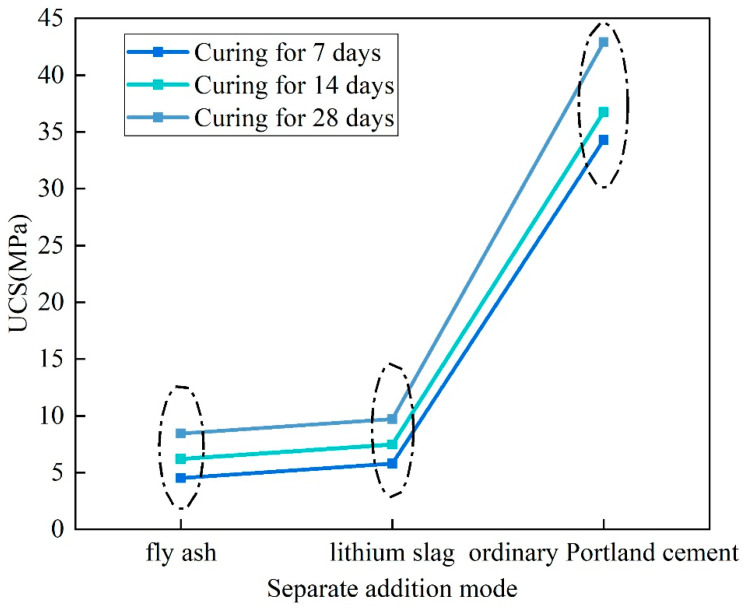
UCS results of backfill specimens with OPC, LS, and FA added separately.

The water–binder ratio of the backfill sample was fixed at 0.6. The compressive strengths of the OPC, LS, and FA binders at different curing ages are displayed in Figure 7. The UCS of the OPC sample is much higher than that of the LS and FA samples. The UCS values of the OPC sample at 7, 14, and 28 days are 34.29, 36.74, and 42.9 MPa, respectively. The UCS of the LS sample is slightly higher than that of the FA sample. The UCS values at curing ages of 7, 14, and 28 days are, respectively, −5.8, 7.46, and 9.71 MPa for the LS sample and −4.51, 6.2, and 8.43 MPa for the FA sample. This shows that the OPC cementing performance is higher than that of LS, but the cementing performance of LS is higher than that of FA.

**Figure 8:**
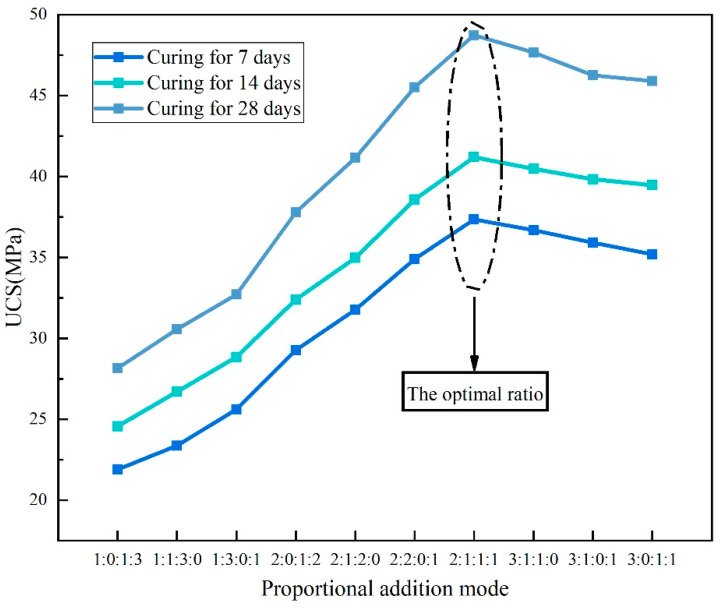
UCS results of backfill specimens with different ratios of OPC, LS, FA, and DG.

The UCS values of the backfill samples with different proportions of OPC, LS, FA, and DG are shown in Figure 8. We can observe the following: (1) With an increase in the content of LS, FA, and DG, the UCS values of all samples exhibit a trend of increasing first and then decreasing. It is worth noting that the compressive strength of the backfilling samples with a total content of LS, FA, and DG less than or equal to 60% in the binder is greater than or close to that of the samples with OPC as the cementitious material. (2) The backfilling sample with an OPC:LS:FA:DG ratio of 2:1:1:1 has the highest compressive strength. The UCS values at 7, 14, and 28 days of curing age are 37.35, 41.2, and 48.73 MPa, respectively, which are 8.9, 12.1, and 13.6% higher than the UCS values of the sample added only with OPC. This is because LS has finer particles that can effectively fill the cracks between larger particles, thereby improving the overall compactness of the filling material. Second, it can be found from the XRD test that LS, FA, and DG contain substances with pozzolanic activity that generate certain hydration products through a reaction with water, which help to improve the compressive strength of the filling material [41,42]. Therefore, the LSB was formulated with an OPC:LS:FA:DG ratio of 2:1:1:1.

**Figure 9:**
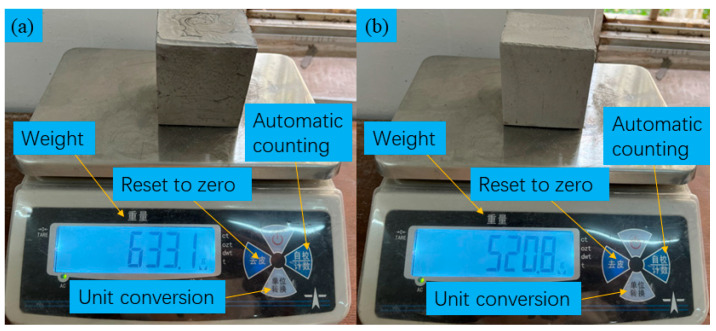
Weight of test blocks using different cementitious materials: (**a**) OPC and (**b**) LSB.

We individually determined the weights of the backfill samples employing LSB and OPC binders, as illustrated in Figure 9. The backfill samples incorporating the LSB binder are approximately 110 g lighter than those utilizing the OPC binder, which is equivalent to a weight reduction of approximately 18%. The observed phenomenon can be attributed to the fact that under the same quality of binder, nearly 60% of the OPC in the LSB-based binder is substituted by LS, FA, and DG materials in the LSB-based binder. These materials, LS, FA, and DG, possess a lower density compared to the OPC material [43]. Furthermore, a SEM sample analysis and grayscale evaluation reveal that the particles in the LSB-based binder, which contribute to the cementing function, are more readily and rapidly involved in hydration reactions. This process leads to the consumption and formation of more lightweight hydrated products, consequently reducing the overall mixed density [44]. Therefore, the LSB-based backfill materials possess a relatively lower self-weight, which can effectively alleviate the load pressure of the backfill on underground mines, reduce the stress on underground structures, diminish surface subsidence, and enhance the safety of mineral extraction. 

### 3.2. LSB–CLTB 

#### 3.2.1. UCS Test Results

**Figure 10:**
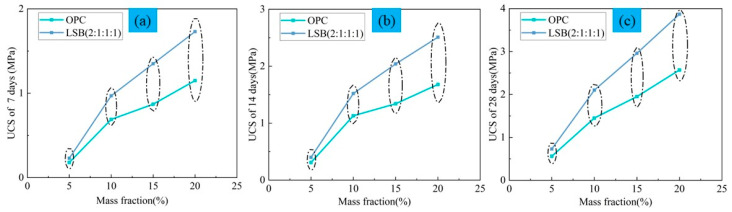
Compressive strength of cemented LFT with different mass fractions and curing ages: (**a**) 7, (**b**) 14, and (**c**) 28 days.

The compressive strength changes of LFT samples cemented with binders with different mass fractions (5, 10, 15, and 20%) of LSB are depicted in Figure 10. The diagram shows the following: (1) The UCS value of the CLTB increases with an increase in the LSB mass fraction at each curing age. Moreover, the compressive properties of all samples using LSB at different curing ages are better than those using OPC. With the increasing mass fraction of the binder, the UCS value of the CLTB increases. The UCS values are approximately 30% and 55% higher than those of the samples using 5 wt% and 20 wt% OPC, respectively. This indicates that LSB is helpful for improving the compressive strength of CLTB. (2) The continuous upgrading and improvement of mining equipment have accelerated the mining speed. The required UCS values of the CLFB in the Yichun Lithium Mine, China, are 0.8, 1.2, and 2 MPa at 7, 14, and 28 days, respectively. In this study, the UCS values achieved with 10% LSB content at each curing age are 0.97, 1.52, and 2.1 MPa, respectively, which all meet the requirements. However, the CLTB with a 10 wt% content of OPC could not satisfy these requirements (0.69, 1.13, and 1.45 MPa).

#### 3.2.2. Mechanism Analysis

**Figure 11:**
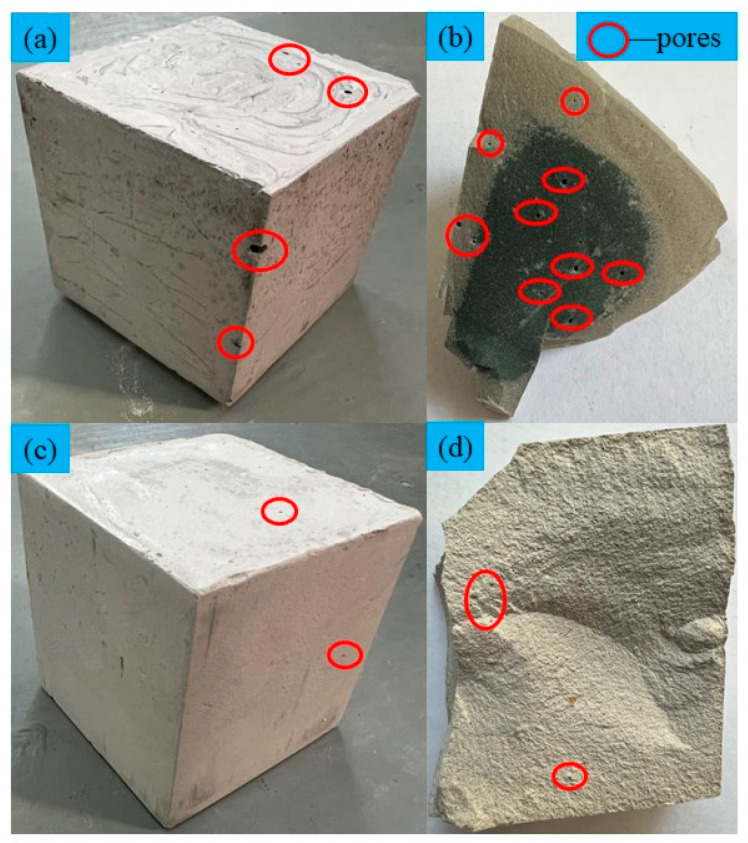
Macroscopic pore diagram of CLTB specimens with different binders: (**a**) external and (**b**) internal pores of OPC-CLTB test block; (**c**) external and (**d**) internal pores of LSB-CLTB test block.

In Figure 11, it is evident that the backfill utilizing OPC-cemented LFT generates many large pores, both on the surface and internally. In contrast, the backfill using the LSB-cemented LFT displays a markedly lower number of small pores. The presence and formation of these pores can have detrimental effects on the strength of CLTB [45]. Previous studies have established that LS exhibits higher water absorption because of its smaller particle size and larger specific surface area [46,47]. The LS used in this study was tested using a fully automatic BET analyzer (Micromeritics ASAP 2460, Mac Instruments Inc., Sandusky, OH, USA), with nitrogen as the adsorption gas. The testing temperature ranged from 77.337 K to 77.359 K, and the degassing period was 8 h. The specific surface area of LS was determined to be 933.45 ± 24.7 m^2^/kg. 

This indicates that the LS used in this study has good surface activity and adsorption capacity. The water absorption of the LS–OPC and OPC pastes was measured. When the LS contents were 20, 30, and 50%, the water absorption of the LS–OPC paste was 30.5, 48.3, and 96.7% higher than that of the OPC paste, respectively. This shows that when using LSB-cemented LFT, the LS particles adsorb a large amount of water in the CLTB slurry, which can provide the required water for the subsequent continuous hydration reaction of OPC and other materials and also reduce the pores generated by water evaporation. Second, by comparing the particle size of each material in the CLTB, it can be seen that LS has the smallest particle size in the slurry, which can sufficiently fill the pores between large particles, such as FA and DG. This makes the internal structure of the CLTB slurry more compact, which is conducive to improving its compressive strength [48]. 

**Figure 12:**
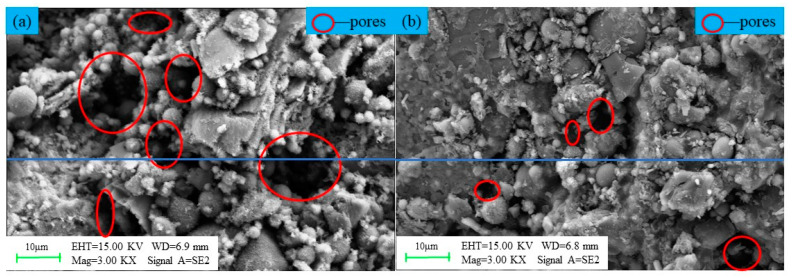
SEM micrographs of CLTB specimens with different binders: (**a**) OPC–CLTB and (**b**) LSB–CLTB specimens.

**Figure 13:**
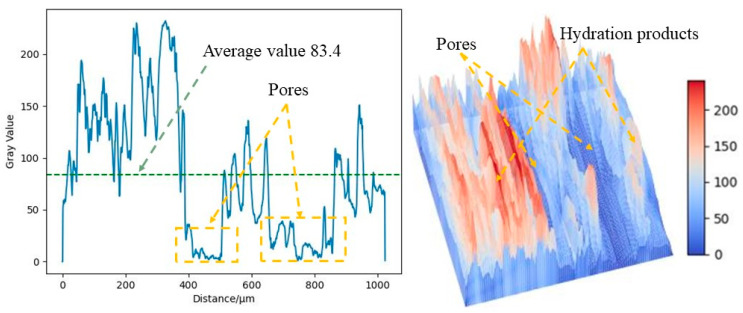
OPC–CLTB gray analysis image.

**Figure 14:**
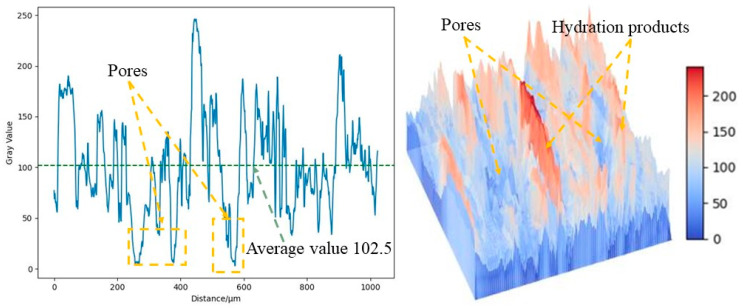
LSB–CLTB gray analysis image.

As shown in Figure 12, the SEM test of the OPC-based CLTB sample reveals that its internal pores are relatively large, whereas those of the LSB–CLTB sample are relatively small, resulting in an overall denser structure. To quantify the density of the qualitative materials more scientifically, a gray value analysis was conducted on the region, and the results are presented in Figure 13 and Figure 14. The average gray value for the OPC-based CLTB sample is 83.4, with pore areas (areas with gray values less than 50) corresponding to spans of 385–503 μm and 654–857 μm on the abscissa axis. In contrast, the LSB-based CLTB sample has an average gray value of 102.5, with pore areas corresponding to spans of 244–283 μm and 557–582 μm on the abscissa axis.

Clearly, the average gray value of the LSB–CLTB sample is higher, and the span of the abscissa corresponding to the pore area is smaller, which can also be more intuitively reflected in the three-dimensional image rendered based on gray value conversion. This is because the uniform distribution of the components in the sample and its relatively dense regional distribution reduce the differences in the interactions between the different components when the SEM electron beam passes through it. Consequently, in areas with fewer pores and better compactness, the electron beam passes through less air or smaller, looser areas, resulting in reduced scattering and transmission. Therefore, it exhibits a higher gray value [49]. This demonstrates that the microscopic structure of the CLTB samples is consistent with their macroscopic properties, further confirming the superiority of the LSB–CLTB. 

**Figure 15:**
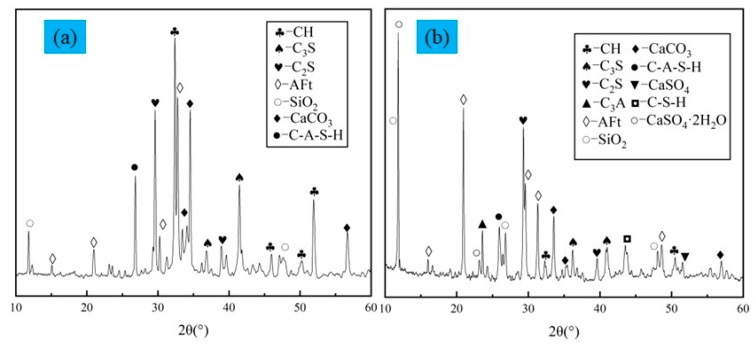
XRD patterns of CLTB samples with different binders: (**a**) OPC–CLTB and (**b**) LSB–CLTB.

In addition, the material contained in the LSB-based binder shows a certain degree of pozzolanic activity in the water addition reaction and produces more stable hydration products. The hydration products further fill the pores between the particles of the backfill material, which improves the compressive strength of the backfill [50,51]. To more clearly and accurately understand the structural properties of the material contained in the sample after the hydration reaction, the OPC and LSB samples (including only the binder and water, excluding LFT) cured for 28 days were tested by XRD, and the results are displayed in Figure 15. In addition to C_3_A, CaSO_4_, and CaSO_4_·2H_2_O in LS and DG in LSB, the LSB and OPC samples show similar phase compositions in their XRD curves.

The hydration reactions of C_3_S, C_2_S, and water contained in OPC, LS, and FA produce a C-S-H gel and CH, and the reaction equations are given in Equations (1) and (2). It can be observed from the XRD results that the OPC sample contains a significant amount of CH. Because of its high porosity, the presence of a large quantity of CH adversely affects its strength [52]. However, in contrast to OPC, LSB contains higher levels of active SiO_2_, Al_2_O_3_, CaO, and C_3_A. C_3_A reacts with water and calcium sulfate to form Aft [53], as shown in Equations (3) and (4). CaO directly reacts with water to produce CH, creating an alkaline environment for subsequent reactions in the backfill system. Meanwhile, SiO_2_ and Al_2_O_3_ with higher reactivity interact with the CH generated in the filling system to form C-S-H, C-A-H, and C-A-S-H gels, and the reaction equations are given in Equations (5)–(7) [54,55,56]. Generally, because these three gels are amorphous and grow together to form a multi-mineral complex gel structure, they cannot be effectively distinguished in the SEM images. By combining the existing research findings with this information, it can be concluded that all three gels exist simultaneously. To facilitate subsequent analyses, we collectively designate them as C-(A)-S-H gels.

Second, under the combined action of DG excitation and the alkaline environment provided by OPC and FA, OH^-^ ions continuously “destroy” the surface of LS and FA particles, leading to dissociation of [SiO_4_]^4−^ and [AlO_4_]^5−^, which releases soluble Si and Al. These ions then interact with Ca^2+^ in the filling system to form hydration products such as an C-S-H gel and AFt. As the hydration products gradually form, they disrupt the equilibrium of aluminate dissolution between the surfaces of the LS and FA particles and the solution, promoting the migration of [AlO_4_]^5−^ from their surfaces. This breaks the connection between [SiO_4_]^4−^ and Al, causing a rapid decrease in the polymerization degree of silicon (aluminum) oxygen tetrahedrons on the surfaces of LS and FA while reactivating the remaining silicon (aluminum) oxygen tetrahedrons. The oligomeric silicate ions released from the dissociated material particles can absorb a certain amount of Ca(OH)_2_ to form a C-(A)-S-H gel and Aft [57]. The interlaced AFt and C-(A)-S-H gel fill the original pores of the backfill, intertwining the material particles and hydration products into a dense internal structure. This significantly improves the mechanical properties of the CLTB [58,59]. Therefore, the LSB matrix with LS, FA, and DG contents less than 60% exhibits higher cementation performance than the OPC matrix, leading to a substantial improvement in the UCS of the CLTB.

**Figure 16:**
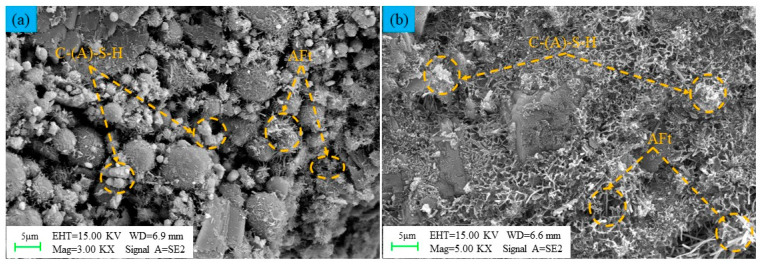
Internal SEM images of LSB–CLTB samples at curing ages of (**a**) 7 and (**b**) 28 days.

As depicted in Figure 16, certain material particle morphologies and pores can be clearly observed in the early stage of the LSB-based CLTB (curing age of 7 days). The surfaces of the particles are gradually filled with needle-like AFt and flocculent C-(A)-S-H gel formed by the hydration reaction. The hydration products effectively fill the pores. In the middle and late stages (curing age of 28 days) of the LSB-based CLTB, with a more thorough hydration reaction, a large number of AFt and C-(A)-S-H gels bind all particles together. These gels continue to grow and diffuse inside the CLTB, resulting in higher compressive strength. The LSB matrix exhibits good pozzolanic activity and can provide good cementing ability for the LFT [60].

(1) $$3CaO\cdot SiO_{2}+2H_{2}O\to 2CaO\cdot SiO_{2}\cdot H_{2}O+Ca{\left(OH\right)}_{2}$$

(2) $$2CaO\cdot SiO_{2}+H_{2}O\to 2CaO\cdot SiO_{2}\cdot H_{2}O$$

(3) $$3CaO\cdot Al_{2}O_{3}+6H_{2}O\to 3CaO\cdot Al_{2}O_{3}\cdot 6H_{2}O$$

(4) $$3CaO\cdot Al_{2}O_{3}\cdot 6H_{2}O+3CaSO_{4}+26H_{2}O\to 3CaO\cdot Al_{2}O_{3}\cdot 3CaSO_{4}\cdot 32H_{2}O$$

(5) $$3Ca{\left(OH\right)}_{2}+SiO_{2}+2H_{2}O\to 3CaO\cdot 2SiO_{2}\cdot 3H_{2}O$$

(6) $$3Ca{\left(OH\right)}_{2}+Al_{2}O_{3}+5H_{2}O\to 3CaO\cdot Al_{2}O_{3}\cdot 6H_{2}O$$

(7) $$2SiO_{2}+Al_{2}O_{3}+4H_{2}O+Ca{\left(OH\right)}_{2}\to Ca{\left(OH\right)}_{2}\cdot Al_{2}O_{3}\cdot 2SiO_{2}\cdot 4H_{2}O$$

### 3.3. Influence of LSB-Cemented LFTs on the Fluidity of Backfill

#### 3.3.1. Results

**Figure 17:**
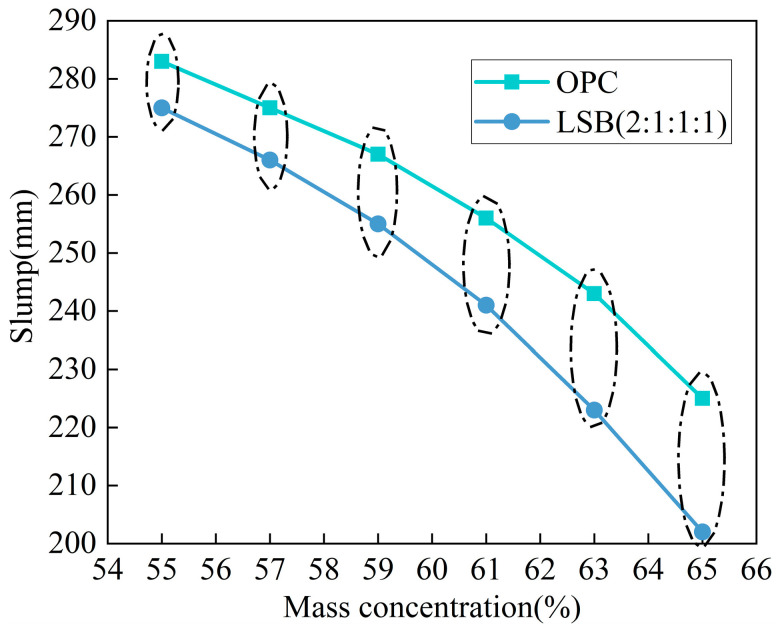
Fluidity of CLTB slurry at different mass concentrations.

The actual backfill conveying pipeline of a lithium mine in Yichun City, China, requires the fluidity of the filling slurry to be higher than 200 mm. In this study, the corresponding mass concentration was 55–65%. Figure 17 shows the fluidity of the CLTB slurry with different mass concentrations of LSB and OPC binders. The following can be observed: (1) As the mass concentration of the CLTB slurry increases from 55% to 65%, the fluidity of the OPC- and LSB-based slurries decreases from 283 mm to 225 mm and from 275 mm to 202 mm, respectively. The overall fluidity reduction value is not significantly different. (2) At the same mass concentration, the fluidity of the LSB–CLTB is lower than that of the OPC–CLTB. (3) Although the LSB binder has an adverse effect on the fluidity of the CLTB slurry, its value is greater than 200 mm, which meets the requirements of the slurry fluidity of the actual filling backfill transportation pipeline at the lithium mine site. This indicates that LSB has a minimal adverse effect on the fluidity and will not substantially affect the transportability of the CLTB [35,36].

#### 3.3.2. Mechanism Analysis

**Figure 18:**
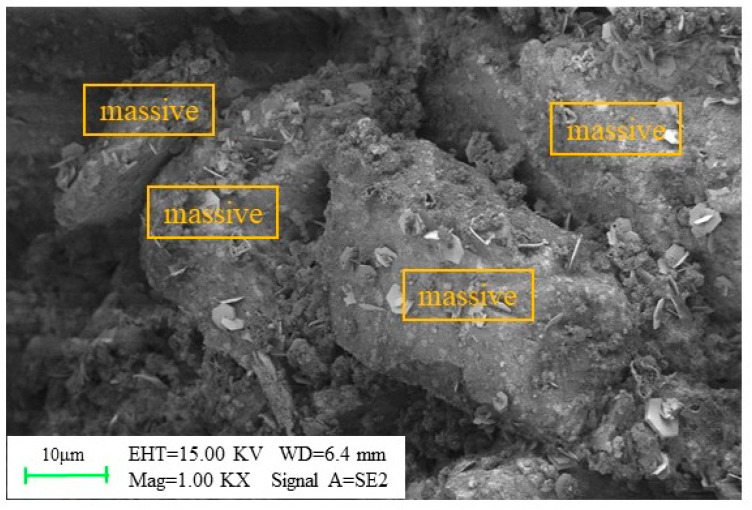
Micromorphology of LSB–CLTB slurry.

The LSB–CLTB paste was examined using SEM, as illustrated in Figure 18. The internal particles of the CLTB exhibit a relatively large “block fiber” structure, which hinders the flow of the CLTB slurry during transportation. This phenomenon is mainly due to the strong water absorption of LS, which causes the particles inside the CLTB slurry to form large block fibers that reduce its fluidity [61]. However, the LSB-based binder has an OPC:LS:FA:DG ratio of 2:1:1:1. This means that in the LSB–CLTB slurry with a mass concentration of 65%, the LS mass fraction is only between 1.2% and 4.3%, according to commonly used cement–sand ratios ranging from 1:10 to 1:2. It should be noted that using 1,200,000 tons of LS solid waste per year can significantly reduce cement consumption by annually utilizing up to 14,400–51,600 tons of mine-discharged LS and other solid wastes, such as FA and DG [18].

### 3.4. Cost–Benefit Analysis

Incorporating the compressive strength results of the LSB-based fillers, the optimal proportions of the LSB-based cementitious material components were determined. A preliminary cost comparison was then conducted with traditional cement-based cementitious materials. An extensive review of the literature and market research revealed the following cost estimates: cement is priced at 460 RMB per ton, fly ash at 180 RMB per ton, desulfurization gypsum at 50 RMB per ton, and lithium slag, which previously cost 100 RMB per ton, experienced a significant price drop, reaching a minimum of 0 RMB per ton. This decline is attributed to the closure of certain lithium carbonate calcination enterprises, among other factors. For the purposes of this cost analysis, a conservative estimate of 50 RMB per ton is adopted for lithium slag [62]. Through the literature review and market research, it is evident that the prices mentioned may vary depending on the region, time, and market conditions. Based on the optimal ratio (2:1:1:1), the unit cost of LSB-based gel materials is calculated as follows: Y = 460 × 40% + 180 × 20% + 50 × 20% + 50 × 20% = 240 RMB. Compared to traditional cement-based materials, each ton of filling gel material can save 220 RMB, which equates to a cost reduction of 47.83% per ton.

## 4. Conclusions

In order to effectively mitigate the environmental issues associated with the storage of solid waste and the high cost of backfill mining presented by traditional cement-based binder fine tailings, this study investigates the feasibility of using an LSB base composed of OPC, LS, FA, and DG as a fundamental binding agent for backfill materials of fine-grained lithium mica tailings. The physicochemical properties of LSB-based materials were characterized using laser particle size analysis, XRD (X-ray diffraction), and XRF (X-ray fluorescence) analyses. In comparison with traditional OPC-CLTB (ordinary Portland cement–lithium mica fine tailings fill), uniaxial compression tests, flowability tests, SEM (Scanning Electron Microscopy), and grayscale value analyses were conducted to investigate and analyze the backfill mechanical performance, microstructural characteristics, and the hydration reaction cementation mechanism of LSB-CLTB. Based on the experimental results, the following conclusions were drawn:

1. The optimal OPC:LS:FA:DG ratio for the LSB is 2:1:1:1. The specimens prepared using this proportion demonstrate significant improvements in their UCS compared with the OPC-based samples. Specifically, after curing for 7, 14, and 28 days, the UCS exhibits increases of 8.9, 12.1, and 13.6%, respectively, relative to those of the OPC-based samples.
2. The LSB shows superior binding capacity when cementing LFTs. Under identical conditions, the LSB–CLTB specimens exhibit higher compressive strengths than the OPC–CLTB specimens. The CLTB prepared with 10 wt% LSB achieves UCS values of 0.97, 1.52, and 2.1 MPa after 7, 14, and 28 days, satisfactorily meeting the strength requirements for backfilling operations at the Yichun Lithium Mine in Jiangxi.
3. The materials within LSB exhibit significant pozzolanic activity. As the curing period increases, the materials with higher levels of pozzolanic activity generate substantial amounts of AFt and C-(A)-S-H gels through hydration reactions, effectively filling the inherent pores within the CLTB. This leads to a denser CLTB, which consequently enhances its mechanical properties. Furthermore, the test results indicate that the fluidity of the LSB-based CLTB slurry satisfies the requirements for actual pipeline transportation in lithium mines.

The findings reveal that the low-cost cemented filling material prepared using LS, FA, and DG combined with LFTs has good working performance and mechanical properties. It also has economic and environmental benefits, as it reduces carbon emissions and utilizes solid waste materials effectively. In future work, the authors will consider further optimization of the proportion of solid waste in LSB-CLTB specimens and more effectively activate the pozzolanic reactivity of the materials. Additionally, efforts will be made to minimize the adverse effects of the materials on the rheological properties of backfill grouts. Finally, a thorough investigation will be conducted on issues encountered in practical applications.

## Figures and Tables

**Table 1:** Physical parameters of lithium slag composite solid waste cementitious material.

Material	d(bar) (μm)	d_10_ (μm)	d_30_ (μm)	d_60_ (μm)	C_u_	C_c_	pH
LS	34.58	4.88	14.94	41.67	8.54	1.1	5.5
DG	53.04	8.9	28.07	77.94	8.76	1.14	7.8
OPC	42.18	9.48	24.1	55.8	5.87	1.1	12.8
FA	97.63	13.77	46.29	141.78	10.3	1.1	11.6
LFT	63.27	11.8	30	100.87	8.55	0.76	6.7

Note: C_u_ = d_60_/d_10_ and C_c_ = (d_30_)^2^/(d_10_ × d_60_). Here, d_10_, d_30_, and d_60_ represent the characteristic particle sizes, whereas d (bar) denotes the average particle size of each material.

**Table 2:** Raw material component properties.

Components		LS	DG	OPC	FA
Chemical composites (%)	SiO_2_	25.79	0.99	23.9	52.16
	Al_2_O_3_	16.84	0.08	8.59	24.2
	CaO	29.17	36.9	52.8	6.4
	Na_2_O	0.28	—	0.46	—
	MgO	0.56	0.84	3.72	1.34
	Fe_2_O_3_	2.68	0.42	4.38	10.87
	K_2_O	3.87	0.02	1	—
	SO_3_	9.75	52.17	4.13	1.3
	LOI	4.58	6.26	1.12	2.8

**Table 3:** Composition of lithium slag composite solid waste binders.

No.	Materials				Water–Binder Ratio	Curing Time
	A:OPC	B:LS	C:FA	D:DG		
1	5	0	0	0	0.6	7 days
2	0	5	0	0		14 days
3	0	0	5	0		28 days
4	1	0	1	3		
5	1	1	3	0		
6	1	3	0	1		
7	2	0	1	2		
8	2	1	2	0		
9	2	2	0	1		
10	2	1	1	1		
11	3	1	1	0		
12	3	1	0	1		
13	3	0	1	1		

## Data Availability

Data are contained within the article.

## Abbreviations

C_3_S	Tricalcium silicate; chemical formula: 3CaO·SiO_2_
C_2_S	Dicalcium silicate; chemical formula: 2CaO·SiO_2_
C_3_A	Tricalcium aluminate; chemical formula: 3CaO·Al_2_O_3_
C-S-H	Calcium silicate hydrate gel; chemical formula: CaO_X_·SiO_2_·(H_2_O)_y_
C-A-H	Calcium aluminate hydrate gel; chemical formula: CaO_X_·Al_2_O_3_·(H_2_O)_y_
C-A-S-H	Calcium silico-aluminate hydrate gel; chemical formula: CaO_X_·(Al_2_O_3_)_y_·SiO_2_·(H_2_O)_z_
AFt	Trisulfate hydrate of calcium sulfoaluminate; chemical formula: 3CaO·Al_2_O_3_·3CASO_4_·32H_2_O
CH	Calcium hydroxide; chemical formula: Ca(OH)_2_
OPC	Cement; the cement referred to in this paper specifically denotes the PO42.5 grade.
LSB	Lithium slag composite solid waste cementitious material
LSB-CLFT	LSB-cemented lithium mica fine tailings fill material
OPC-CLFT	OPC-cemented lithium mica fine tailings fill
LS	Lithium slag
FA	Fly ash
DG	Desulfurization gypsum
LFT	Lithium mica fine tailings

## References

[1] Gao T.M., Fan N., Chen W., Dai T. (2023). Lithium extraction from hard rock lithium ores (spodumene, lepidolite, zinnwaldite, petalite): Technology, resources, environment and cost. China Geol..

[2] Shahjalal M., Roy P.K., Shams T., Fly A., Chowdhury J.I., Ahmed R., Liu K. (2022). A review on second-life of Li-ion batteries: Prospects, challenges, and issues. Energy.

[3] Kelly J.C., Wang M., Dai Q., Winjobi O. (2021). Energy, greenhouse gas, and water life cycle analysis of lithium carbonate and lithium hydroxide monohydrate from brine and ore resources and their use in lithium ion battery cathodes and lithium ion batteries. Resour. Conserv. Recycl..

[4] Xiao E., Krumins V., Tang S., Xiao T., Ning Z., Lan X., Sun W. (2016). Correlating microbial community profiles with geochemical conditions in a watershed heavily contaminated by an antimony tailing pond. Environ. Pollut..

[5] He X., Li W., Yang J., Su Y., Zhang Y., Zeng J., Dai F., Tan H. (2023). Multi-solid waste collaborative production of clinker-free cemented iron tailings backfill material with ultra-low binder-tailing ratio. Constr. Build. Mater..

[6] Kou Y., Deng Y., Tan Y., Han C., Song W. (2023). Hydration Characteristics and Early Strength Evolution of Classified Fine Tailings Cemented Backfill. Materials.

[7] Qi C., Fourie A. (2019). Cemented paste backfill for mineral tailings management: Review and future perspectives. Miner. Eng..

[8] Kinnunen P.H.M., Kaksonen A.H. (2019). Towards circular economy in mining: Opportunities and bottlenecks for tailings valorization. J. Clean. Prod..

[9] Peng X., Guo L., Liu G., Yang X., Chen X. (2021). Experimental Study on Factors Influencing the Strength Distribution of In Situ Cemented Tailings Backfill. Metals.

[10] Tariq A., Yanful E.K. (2013). A review of binders used in cemented paste tailings for underground and surface disposal practices. J. Environ. Manag..

[11] Behera S.K., Ghosh C.N., Mishra D.P., Singh P., Mishra K., Buragohain J., Mandal P.K. (2020). Strength development and microstructural investigation of lead-zinc mill tailings based paste backfill with fly ash as alternative binder. Cem. Concr. Compos..

[12] Chen S.C., Gao M.Y., Lin W.T., Liang J.F., Li D.W. (2022). Effects of incorporating large quantities of copper tailings with various particle sizes on the strength and pore structure of cement-based materials. Constr. Build. Mater..

[13] Qiu J., Guo Z., Yang L., Jiang H., Zhao Y. (2020). Effect of tailings fineness on flow, strength, ultrasonic and microstructure characteristics of cemented paste backfill. Constr. Build. Mater..

[14] Zhao K., Zhou Y., Huang Q., Yin S., Yan Y., Wu J., Shen L., Zeng X., Liu W. (2023). Early properties and modeling of cemented superfine tailings backfill containing sodium dodecyl sulfate: Microstructure, mechanics, and acoustics. Mech. Mater..

[15] Andrew R.M. (2018). Global CO_2_ emissions from cement production. Earth Syst. Sci. Data.

[16] Santana-Carrillo J.L., Burciaga-Díaz O., Escalante-Garcia J.I. (2022). Blended limestone-Portland cement binders enhanced by waste glass based and commercial sodium silicate-Effect on properties and CO_2_ emissions. Cem. Concr. Compos..

[17] Chen D., Hu X., Shi L., Cui Q., Wang H., Yao H. (2012). Synthesis and characterization of zeolite X from lithium slag. Appl. Clay Sci..

[18] Ding Z., Ma W., Wei K., Wu J., Zhou Y., Xie K. (2012). Boron removal from metallurgical-grade silicon using lithium containing slag. J. Non-Cryst. Solids.

[19] Ayati B., Newport D., Wong H., Cheeseman C. (2022). Acid activated smectite clay as pozzolanic supplementary cementitious material. Cem. Concr. Res..

[20] Filho J.H., Medeiros MH F., Pereira E., Helene P., Isaia G.C. (2013). High-volume fly ash concrete with and without hydrated lime: Chloride diffusion coefficient from accelerated test. J. Mater. Civ. Eng..

[21] Janardhan P., Krishnaiah V. (2022). An improved mechanical behaviour of fly ash and GGBS based geopolymer concrete. Mater. Today Proc..

[22] Wan Y., Hui X., He X., Xue J., Feng D., Chen Z., Li J., Liu L., Xue Q. (2022). Utilization of flue gas desulfurization gypsum to produce green binder for dredged soil solidification: Strength, durability, and planting performance. J. Clean. Prod..

[23] Jiang L., Li C., Wang C., Xu N., Chu H. (2018). Utilization of flue gas desulfurization gypsum as an activation agent for high-volume slag concrete. J. Clean. Prod..

[24] Wang X., Yu Y., Zou F., Luo H., Zhou Z., Zhu J., Guo G., Zhong Y. (2023). High performance CASH seeds from fly ash-carbide slag for activating lithium slag towards a low carbon binder. J. Environ. Manag..

[25] Yoon J., Jafari K., Tokpatayeva R., Peethamparan S., Olek J., Rajabipour F. (2022). Characterization and quantification of the pozzolanic reactivity of natural and non-conventional pozzolans. Cem. Concr. Compos..

[26] Liu T., Shi G., Li G., Wang Z. (2019). Study on properties of foamed concrete with EPS as coarse aggregate. IOP Conf. Ser. Mater. Sci. Eng..

[27] Qiu W., Hu W., Curtin D., Motoi L. (2021). Soil particle size range correction for improved calibration relationship between the laser-diffraction method and sieve-pipette method. Pedosphere.

[28] Xue G., Yilmaz E., Song W., Cao S. (2020). Fiber length effect on strength properties of polypropylene fiber reinforced cemented tailings backfill specimens with different sizes. Constr. Build. Mater..

[29] Liu Z., Wang J., Jiang Q., Cheng G., Li L., Kang Y., Wang D. (2019). A green route to sustainable alkali-activated materials by heat and chemical activation of lithium slag. J. Clean. Prod..

[30] Rahman S.M.A., Dodd A., Khair S., Shaikh F.U., Sarker P.K., Hosan A. (2023). Assessment of lithium slag as a supplementary cementitious material: Pozzolanic activity and microstructure development. Cem. Concr. Compos..

[31] Aldhafeeri Z., Fall M. (2017). Sulphate induced changes in the reactivity of cemented tailings backfill. Int. J. Miner. Process..

[32] Feng Y., Kero J., Yang Q., Chen Q., Engström F., Samuelsson C., Qi C. (2019). Mechanical activation of granulated copper slag and its influence on hydration heat and compressive strength of blended cement. Materials.

[33] Zhang Q., Chen Q., Wang X. (2016). Cemented backfilling technology of paste-like based on aeolian sand and tailings. Minerals.

[34] (2005). Test Method for Flow of Cement Mortar.

[35] Chen Q., Zhang Q., Fourie A., Xin C. (2017). Utilization of phosphogypsum and phosphate tailings for cemented paste backfill. J. Environ. Manag..

[36] Qi C., Chen Q., Fourie A., Qi C., Chen Q., Fourie A., Zhao J., Zhang Q. (2018). Pressure drop in pipe flow of cemented paste backfill: Experimental and modeling study. Powder Technol..

[37] Xiu Z., Wang S., Ji Y., Wang F., Ren F., Nguyen V.T. (2021). Loading rate effect on the uniaxial compressive strength (UCS) behavior of cemented paste backfill (CPB). Constr. Build. Mater..

[38] Zhou S., Li X., Zhou Y., Min C., Shi Y. (2020). Effect of phosphorus on the properties of phosphogypsum-based cemented backfill. J. Hazard. Mater..

[39] Feng Y., Yang Q., Chen Q., Kero J., Andersson A., Ahmed H., Engström F., Samuelsson C. (2019). Characterization and evaluation of the pozzolanic activity of granulated copper slag modified with CaO. J. Clean. Prod..

[40] Niroshan N., Yin L., Sivakugan N., Veenstra R.L. (2018). Relevance of SEM to long-term mechanical properties of cemented paste backfill. Geotech. Geol. Eng..

[41] Wu P., Wang J., Hu S., Cao X., Lyu X. (2018). Preparation and performance of slag-based binders for the cementation of fine tailings. J. Adhes. Sci. Technol..

[42] Hu Y., Ren X., Ye J., Luan Z., Xiao Y., Zhang W. (2023). Performance of a steel slag-based supplementary cementitious material via the synergistic modification of binary fly ash and gypsum system. Constr. Build. Mater..

[43] Li J., Yilmaz E., Cao S. (2020). Influence of solid content, cement/tailings ratio, and curing time on rheology and strength of cemented tailings backfill. Minerals.

[44] Allen A.J., Thomas J.J., Jennings H.M. (2007). Composition and density of nanoscale calcium–silicate–hydrate in cement. Nat. Mater..

[45] He Y., Chen Q., Qi C., Zhang Q., Xiao C. (2019). Lithium slag and fly ash-based binder for cemented fine tailings backfill. J. Environ. Manag..

[46] Wu F.F., Shi K.B., Dong S.K. (2014). Properties and microstructure of HPC with lithium-slag and fly ash. Key Eng. Mater..

[47] Wu F.F., Shi K.B., Dong S.K. (2014). Influence of concrete with lithium-slag and steel slag by early curing conditions. Key Eng. Mater..

[48] Huang Z., Cao S., Yilmaz E. (2023). Microstructure and mechanical behavior of cemented gold/tungsten mine tailings-crushed rock backfill: Effects of rock gradation and content. J. Environ. Manag..

[49] Mendoza F., Lu R. (2015). Basics of image analysis. Hyperspectral Imaging Technology in Food and Agriculture.

[50] Zheng J., Sun X., Guo L., Zhang S., Chen J. (2019). Strength and hydration products of cemented paste backfill from sulphide-rich tailings using reactive MgO-activated slag as a binder. Constr. Build. Mater..

[51] Wang J., Fu J., Song W., Zhang Y. (2022). Effect of rice husk ash (RHA) dosage on pore structural and mechanical properties of cemented paste backfill. J. Mater. Res. Technol..

[52] Cihangir F., Ercikdi B., Kesimal A., Ocak S., Akyol Y. (2018). Effect of sodium-silicate activated slag at different silicate modulus on the strength and microstructural properties of full and coarse sulphidic tailings paste backfill. Constr. Build. Mater..

[53] Zhang Y., Yu P., Pan F., He Y. (2018). The synergistic effect of AFt enhancement and expansion in Portland cement-aluminate cement-FGD gypsum composite cementitious system. Constr. Build. Mater..

[54] Zhang S., Shi T., Ni W., Li K., Gao W., Wang K., Zhang Y. (2021). The mechanism of hydrating and solidifying green mine fill materials using circulating fluidized bed fly ash-slag-based agent. J. Hazard. Mater..

[55] Fu J.X., Wang K., Wang J. (2023). Internal pore evolution and early hydration characterization of fly ash cement backfill. J. Build. Eng..

[56] Yang P., Liu L., Suo Y., Qu H., Xie G., Zhang C., Deng S., Lv Y. (2023). Investigating the synergistic effects of magnesia-coal slag based solid waste cementitious materials and its basic characteristics as a backfill material. Sci. Total Environ..

[57] Mikheenkov M.A., Sheshukov O.Y., Lobanov D.A. (2017). Reduction of environmental pressure by giving cementing material properties to the ferrous slags. KnE Mater. Sci..

[58] Xu S., Xu Z., Ji Y. (2023). Preparation and Mechanical Properties of Low Carbon Cementitious Materials with Superfine Cement Reverse Filling High-Volume Mineral Admixtures. Materials.

[59] Luo Q., Liu Y., Dong B., Ren J., He Y., Wu K., Wang Y. (2023). Lithium slag-based geopolymer synthesized with hybrid solid activators. Constr. Build. Mater..

[60] He Y., Kang Q., Lan M., Peng H. (2023). Mechanism and assessment of the pozzolanic activity of melting-quenching lithium slag modified with MgO. Constr. Build. Mater..

[61] Sarkar G., Siddiqua S. (2016). Effect of fluid chemistry on the microstructure of light backfill: An X-ray CT investigation. Eng. Geol..

[62] Huang Y., Zhao X., Zhang R., Xie P., Xue G., Ma G. (2024). Environmental economic profiles of expressway construction via life cycle assessment. Environ. Impact Assess. Rev..

